# Detection and Genetic Characterization of Canine Adenoviruses, Circoviruses, and Novel Cycloviruses From Wild Carnivores in Italy

**DOI:** 10.3389/fvets.2022.851987

**Published:** 2022-03-31

**Authors:** Linda A. Ndiana, Gianvito Lanave, Violetta Vasinioti, Costantina Desario, Camillo Martino, Maria Loredana Colaianni, Francesco Pellegrini, Antonio Camarda, Shadia Berjaoui, Giovanni Sgroi, Gabriella Elia, Annamaria Pratelli, Francesco Buono, Vito Martella, Canio Buonavoglia, Nicola Decaro

**Affiliations:** ^1^Department of Veterinary Medicine, University of Bari, Bari, Italy; ^2^Istituto Zooprofilattico Sperimentale dell'Abruzzo e del Molise 'G. Caporale', Teramo, Italy; ^3^Istituto Zooprofilattico Sperimentale di Puglia e Basilicata, Foggia, Italy; ^4^Department of Veterinary Medicine and Animal Production, University of Naples Federico II, Naples, Italy

**Keywords:** wild carnivores, canine adenovirus type 1, canine circovirus, cycloviruses, molecular survey

## Abstract

Wild carnivores are known to play a role in the epidemiology of several canine viruses, including canine adenoviruses types 1 (CAdV-1) and 2 (CAdV-2), canine circovirus (CanineCV) and canine distemper virus (CDV). In the present study, we report an epidemiological survey for these viruses in free ranging carnivores from Italy. A total of 262 wild carnivores, including red foxes (*Vulpes vulpes*), wolves (*Canis lupus*) and Eurasian badgers (*Meles meles*) were sampled. Viral nucleic acid was extracted and screened by real-time PCR assays (qPCR) for the presence of CAdVs and CanineCV DNA, as well as for CDV RNA. CAdV-1 DNA was detected only in red foxes (4/232, 1.7%) whilst the wolves (0/8, 0%) and Eurasian badgers (0/22, 0%) tested negative. CanineCV DNA was detected in 4 (18%) Eurasian badgers, 4 (50%) wolves and 0 (0%) red foxes. None of the animals tested positive for CDV or CAdV-2. By sequence and phylogenetic analyses, CAdV-1 and CanineCV sequences from wild carnivores were closely related to reference sequences from domestic dogs and wild carnivores. Surprisingly, two sequences from wolf intestines were identified as cycloviruses with one sequence (145.20-5432) displaying 68.6% nucleotide identity to a cyclovirus detected in a domestic cat, while the other (145.201329) was more closely related (79.4% nucleotide identity) to a cyclovirus sequence from bats. A continuous surveillance in wild carnivores should be carried out in order to monitor the circulation in wildlife of viruses pathogenic for domestic carnivores and endangered wild species.

## Introduction

Several pathogenic viruses of domestic dogs (*Canis lupus familiaris*) possess the ability to infect different species of wild carnivores. This multi-host capacity is frequently observed with canine parvovirus (CPV), canine adenoviruses (CAdVs) and canine circovirus (CanineCV) (1–6). CAdVs are non-enveloped, icosahedral members of the genus *Mastadenovirus* in the family *Adenoviridae*, with a double-stranded DNA genome about 32 kb long. Two distinct types of the virus are known, canine adenovirus type 1 (CAdV-1), and canine adenovirus type 2 (CAdV-2), causing infectious canine hepatitis (ICH) and kennel cough in dogs, respectively (7). CAdV-1 was described in silver foxes (*Vulpes vulpes)* displaying the so-called “epizootic fox encephalitis” in 1930 (8) and has been subsequently detected in diseased and apparently healthy wild canids (1, 9–12). ICH has been extensively controlled by vaccination with occasional reports of re-emergence in domestic dog populations (13–17), as a consequence of importation of infected pups from endemic areas or possible contact with wild canids, due to the propensity of the virus to establish persistent infections in these animals (18).

Unlike CAdVs, CanineCV is a relatively newly discovered virus belonging to the family *Circoviridae* (19). There are two genera under this family, namely *Circovirus* and *Cyclovirus*, with CanineCV belonging to the former. They are DNA viruses with a circular, ambisense, single-stranded genome enclosed in a non-enveloped icosahedral capsid (20). CanineCV has had conflicting reports regarding its role in severe disease of domestic dogs. Notwithstanding its poorly understood pathogenesis, the virus has been associated with systemic or enteric disease in dogs (21–27). CanineCV has been reported in wild carnivores, especially foxes and wolves (6, 28, 29). Similar to what is reported in dogs (30), CanineCV has been suggested to exacerbate the clinical course of other infections in foxes, wolves and badgers (6). In contrast, cycloviruses have been detected in several animal species including birds, chimpanzees, as well as humans (31) but they have not been previously associated with infection of foxes, wolves, and badgers.

CDV (genus *Morbillivirus*, family *Paramyxoviridae*) is non-segmented, negative-stranded RNA genome virus, which is responsible for severe systemic disease in dogs, characterized by a variety of clinical signs, including fever, respiratory and enteric signs, and neurological disorders (32). CDV can also infect a wide range of animals including wild canids and felids (33, 34). This virus has been previously and frequently detected in wild carnivores across Italy and poses a threat to animal conservation and re-emergence in domestic dog populations (35, 36).

Interspecies transmission of viruses between wildlife and domestic dogs occurs, especially at the human-animal interface, impacting both virus evolution and epidemiology (28), and sometimes resulting in epizootics. In the present study, we describe an epidemiological survey for selected viruses of dogs, including canine adenoviruses, circoviruses, and canine distemper virus, in wild carnivores in Italy.

## Materials and Methods

### Sample Collection and Nucleic Acid Extraction

A total of 262 samples consisting of spleens (*n* = 255) and intestines (*n* = 7) were collected from wild animals found dead, including foxes (*Vulpes vulpes*) (*n* = 232), wolves (*Canis lupus*) (*n* = 8), and Eurasian badgers (*Meles meles*) (*n* = 22). Sampling was carried out in different regions of central and southern Italy including Tuscany, Abruzzi, Lazio, Molise, Campania, Calabria, Apulia and Basilicata from 2014 to 2020 (4).

Viral nucleic acid was extracted from 200 μl of the supernatants of samples homogenized in minimum essential medium (10% w/v), using the QIAmp Cador Pathogen Mini Kit (Qiagen S.p.A., Milan, Italy), following the manufacturer's protocol.

### Screening for Canine Viruses

Screening of nucleic acid extracts was carried out using real-time reverse transcriptase-PCR (RT-qPCR) assays. CAdVs were detected using a CAdV-specific primer pair and virus-specific probes to discriminate between CAdV-1 and CAdV-2 (37) (Supplementary Table 1), whilst CanineCV were searched for with specific primers and probe targeting the Rep encoding gene of CanineCV (26). These TaqMan-based RT-PCR assays were carried out using iTaq™ Universal Probes Supermix (Bio-Rad Laboratories Srl, Milan, Italy) in a final volume of 50 μl consisting of 25 μl of Supermix, 600 nM of forward and reverse primers, 400 nM of probe and 20 μl of nucleic acid extracts. The thermal protocol for CAdV and CanineCV was as follows: activation of iTaq DNA polymerase at 95°C for 10 min and 45 cycles of denaturation at 95°C for 15 s and annealing/extension at 60°C for 1 min.

Extracts were reverse transcribed prior to testing for CDV following an earlier described qPCR protocol (38). In brief, the reaction volume of 20 μl for synthesis of c-DNA consisted of PCR buffer 1×, MgCl2 5 mM, 1 mM of deoxynucleotide, RNase Inhibitor 1 U, reverse transcriptase 2.5 U, random hexamers 2.5 U. Reverse transcription was run under the following cycling conditions: 42°C for 30 min, followed by a denaturation step at 99°C for 5 min. Real-time PCR mix was prepared with the same reagents and concentrations of CAdV and CanineCV. The thermal protocol consisted of activation of iTaq DNA polymerase at 95°C for 10 min and 45 cycles of denaturation at 95°C for 15 s, primer annealing at 48°C for 1 min and extension at 60°C for 1 min.

### PCR Amplification

For all CanineCV-positive samples, amplification of partial rolling circle replication initiator protein (Rep) gene (400 bp) was performed by a nested PCR, using consensus primers CV-F1/CV-R1 and CV-F2/CV-R2 (31) (Supplementary Table 1). Overlapping segments of CAdV hexon gene were also amplified from positive samples using primer pairs CAV-F/HEX-R and HEX-F/CAV-R (Supplementary Table 1), yielding overlapping fragments of 1,882 bp and 1,009 bp, respectively. The PCR assay, performed in a final volume of 50 μl, contained 5 μl of DNA extract, TaKaRa LA TaqTM Kit (Takara Bio Europe S.A.S. Saint-Germain-en-Laye, France) consisting of 24.5 μl of PCR grade water, 5 μl of 10x buffer, 5 μl of MgCl2 (25 mM), 900 nmol/L of forward and reverse primers, 8 μl of deoxynucleotides. Cycling conditions included an initial denaturation at 94°C for 2 min, 35 cycles consisting of 30 s of denaturation, 30 s of annealing and 3 min of extension at 94, 58, and 68°C, respectively, followed by a final extension at 72°C for 10 min.

### Sequence and Phylogenetic Analyses

All PCR products were purified using Qiaquick PCR purification Kit (Qiagen GmbH, Hilden, Germany). PCR products were sequenced in both directions using classical dideoxy Sanger sequencing with BigDye 3.1 Ready Reaction Mix (Applied Biosystems), following the manufacturer's instructions. Sequence reads were assembled using Geneious Prime® 2021.2.2 (https://www.geneious.com). Analyses of the sequences with web-based tools BLAST (https://blast.ncbi.nlm.nih.gov/Blast.cgi?PAGE_TYPE=BlastSearch) and FASTA (https://www.ebi.ac.uk/Tools/sss/fasta/nucleotide.html) with default values were used to find homologous hits. The obtained CAdV sequences were aligned with cognate CAdV and bat adenovirus (used as outgroup) sequences retrieved from the GenBank database by MAFFT algorithm (39). The obtained CanineCV and CV sequences were aligned with cognate circovirus and cyclovirus sequences, respectively, retrieved from the GenBank database by MAFFT algorithm (39).

Phylogenetic analyses were performed with Bayesian inference by MrBayes software using 4 chains run for >1 million generations (40, 41) and Model Test software (http://evomics.org/resources/software/molecular-evolutionsoftware/modeltest/) was used to identify the most appropriate model of evolution for the entire dataset and for each gene individually. The identified program settings for all partitions, under the Akaike Information Criteria, included 2-character states (Hasegawa–Kishino–Yano model) and a proportion of invariable sites.

### GenBank Sequence Submission

The obtained sequences were deposited in the GenBank database under accession numbers OL323110, OL323111, OL323112 and OL323113 for adenoviruses, OL364172, OL364173, OL364174, and OL638989 for circoviruses, and OL638987 and OL638988 for cycloviruses.

## Results

**Figure 1:**
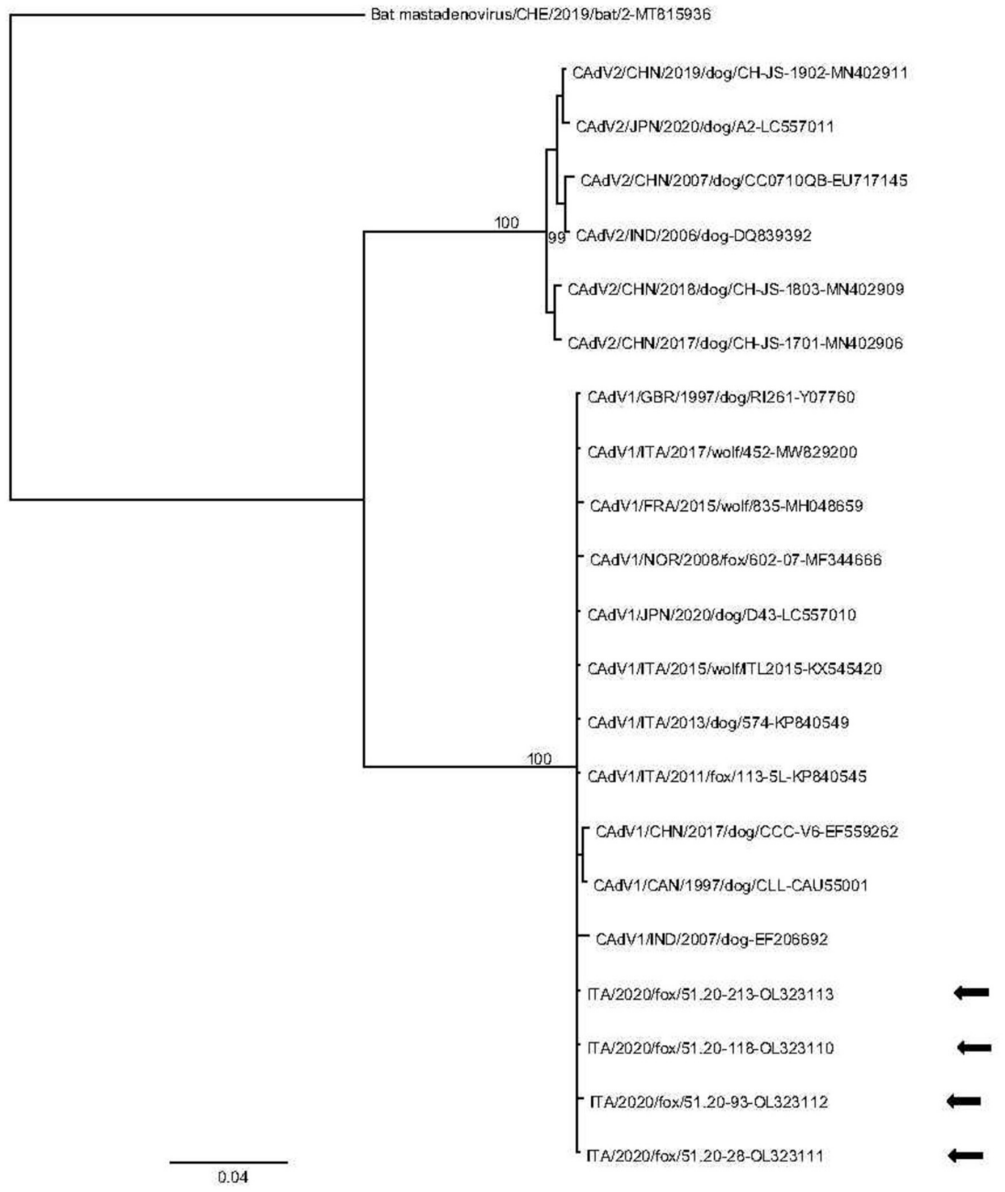
Phylogenetic tree based on alignment of the partial (848 nt) hexon gene of canine adenovirus (CAdV) sequences identified in this study and retrieved from the GenBank database. Bat mastadnovirus (GenBank accession no. MT815936) was used as outgroup. Posterior output of the tree was derived from Bayesian inference using 4 chains run for >1 million generations, 2-character states (Hasegawa–Kishino–Yano) model, a proportion of invariable sites and a subsampling frequency of 1,000. Posterior probability values >95 are indicated on the tree nodes. The black arrows indicate the sequences identified in this study. Scale bar indicates nucleotide substitutions per site.

The results of the molecular screening for the selected canine viruses are reported in Table 1. CAdV-1 DNA was detected in red foxes (4/232, 1.7%), while no wolves (0/8, 0%) nor Eurasian badgers (0/22, 0%) tested positive. Out of the 4 CAdV-1-positive samples, 2 whole (OL323111 and OL323112) and two partial (OL323110 and OL323113) hexon gene sequences were generated and analyzed. Blast analysis showed 99.89 to 100% identity between the sequences from this study and 99.21–100 % identity with CAdV-1 reference sequences obtained from domestic dogs in Italy and Japan. Phylogenetic analyses of CAdVs from this study and other reference sequences from GenBank revealed a distinct distribution of the analyzed sequences into two clades, consisting of CAdV-1 and CAdV-2, respectively (Figure 1). Hexon gene sequences were nearly identical to those previously generated from other wild animals and domestic dogs with only three synonymous substitutions observed in viral sequence 51.20-28 (OL323111) at position 1356 (C to A) and sequence 51.20-93 (OL323112), displaying changes from C to T at positions 372 and 2,241 compared to reference sequence 574-2013-RS (KP840549) obtained from a dog in Italy in 2013.

**Table 1:** Sample distribution according to Italian regions, year of collection and wild carnivore species.

**Animal species**	**Year**	**Region**	**Prot. No**.	**Tissue**	**CAdV-1**	**CanineCV**	**CV**	***Carnivore***
								***protoparvovirus* 1^a^**
Eurasian badger	2020	Campania	136.20-6	spleen	neg	**pos**	neg	neg
Eurasian Badger	2020	Campania	136.20-8	spleen	neg	**pos**	neg	neg
Eurasian badger	2020	Campania	136.20-11	spleen	neg	**pos**	neg	neg
Eurasian Badger	2020	Campania	136.20-12	spleen	neg	**pos**	neg	neg
Wolf	2020	Abruzzi	145.20-1274	intestine	neg	**pos**	neg	CPV-2b
Wolf	2020	Abruzzi	145.20-1329	intestine	neg	**pos**	**pos**	neg
Wolf	2020	Abruzzi	145.20-4615	intestine	neg	**pos**	neg	CPV-2b
Wolf	2020	Abruzzi	145.20-5432	intestine	neg	**pos**	**pos**	neg
Fox	2014	Campania	51.20-28	spleen	**pos**	neg	neg	neg
Fox	2014	Campania	51.20-93	spleen	**pos**	neg	neg	neg
Fox	2014	Campania	51.20-118	spleen	**pos**	neg	neg	neg
Fox	2017	Calabria	51.20-213	spleen	**pos**	neg	neg	neg

a *Ndiana et al. (4)*.

*CAdV-1, canine adenovirus type 1; CanineCV, canine circovirus; CV, cyclovirus; pos, positive; neg, negative*.

**Figure 2:**
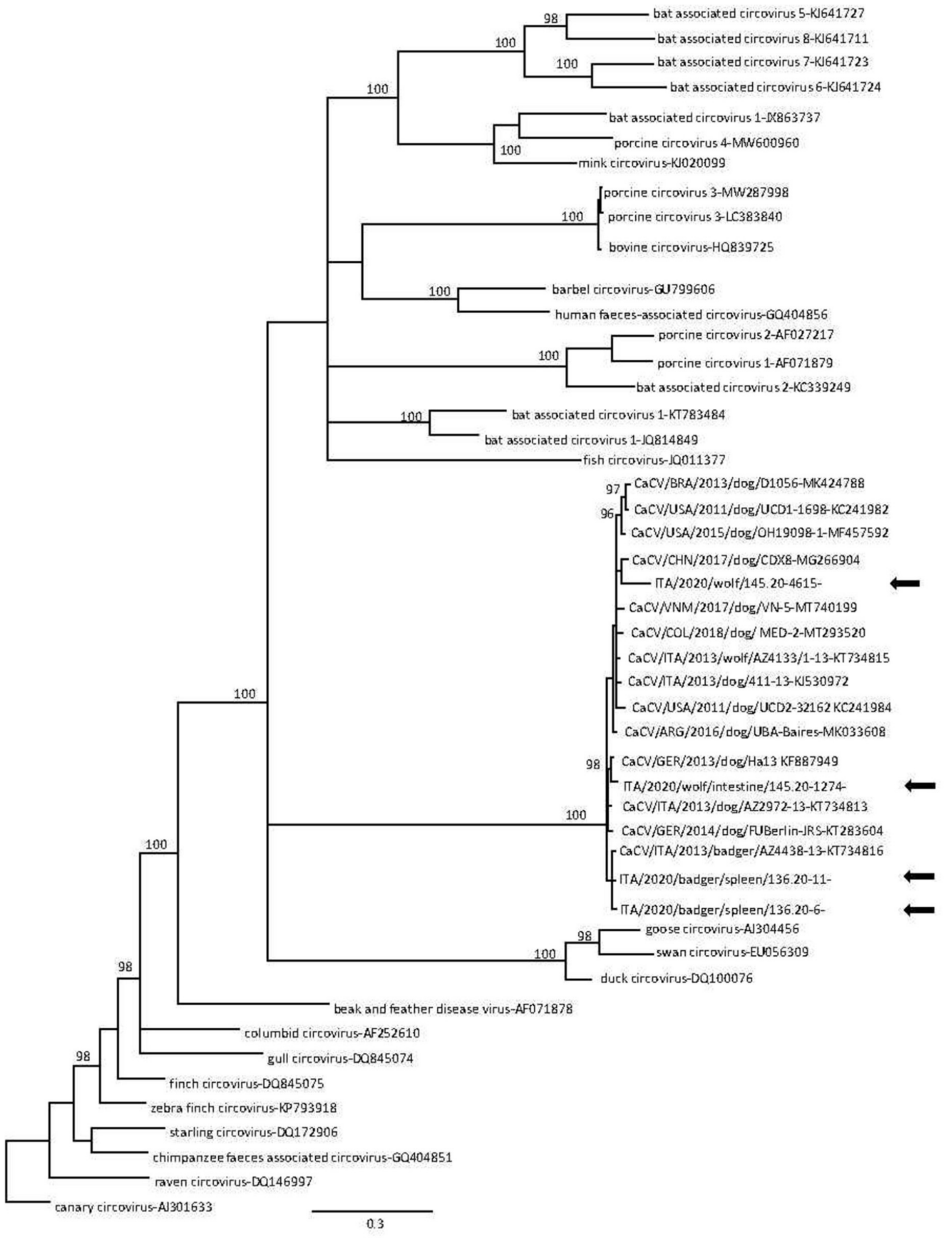
Phylogenetic tree based on alignment of the partial replicase gene (417 nt) of canine circovirus (CanineCV) sequences detected in this study and other circoviruses retrieved from the GenBank database. Canary circovirus (GenBank accession no. AJ301633) was used as outgroup. Posterior output of the tree was derived from Bayesian inference using 4 chains run for >1 million generations, 2-character states (Hasegawa–Kishino–Yano) model, a proportion of invariable sites and a subsampling frequency of 1,000. Posterior probability values >95 are indicated on the tree nodes. The black arrows indicate the sequences identified in this study. Scale bar indicates nucleotide substitutions per site.

**Figure 3:**
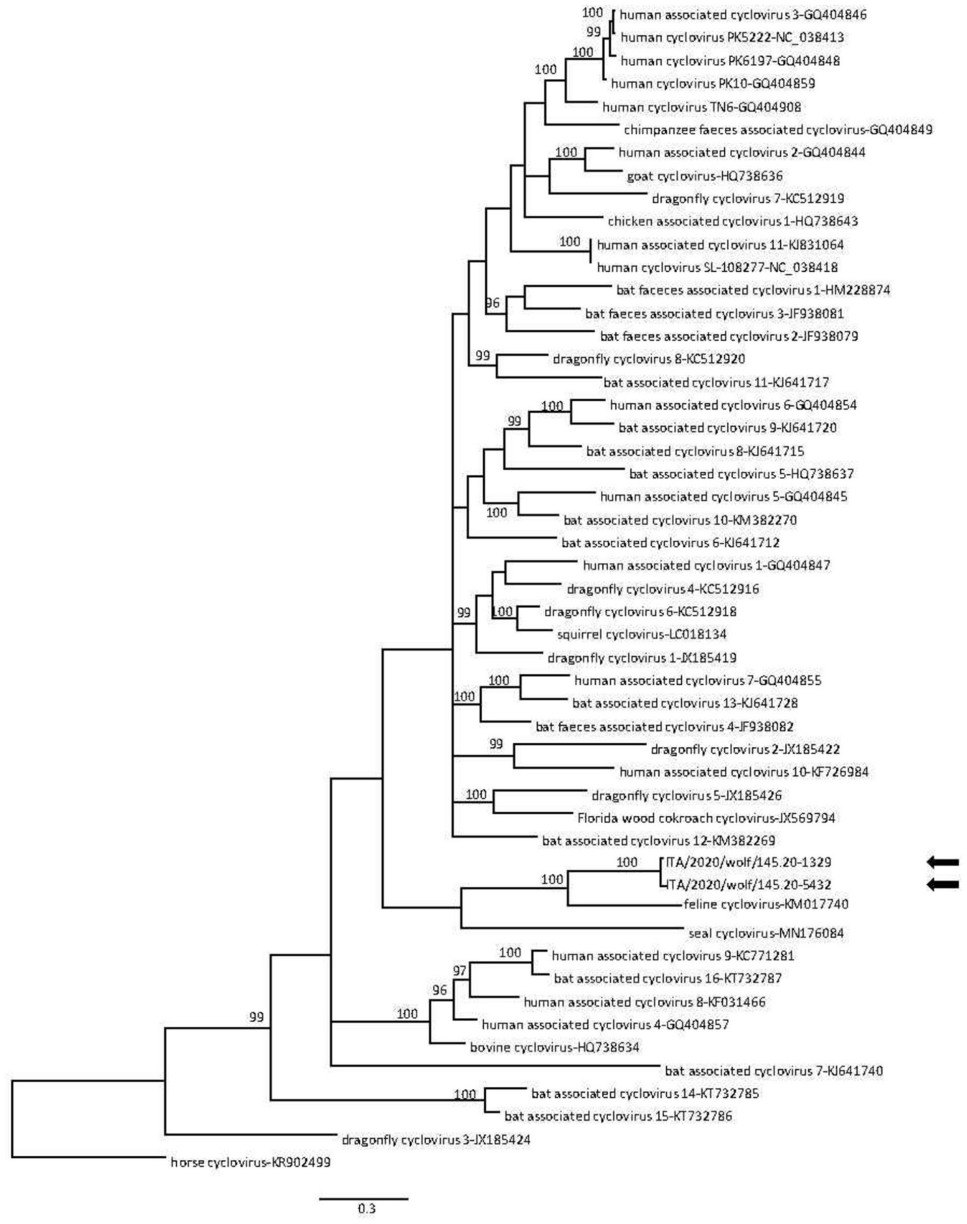
Phylogenetic tree based on alignment of the partial replicase gene (373 nt) of canine cyclovirus isolates detected in this study and other cycloviruses retrieved from the GenBank database. Horse cyclovirus (GenBank accession no. KR902499) was used as outgroup. Posterior output of the tree was derived from Bayesian inference using 4 chains run for >1 million generations, 2-character states (Hasegawa–Kishino–Yano) model, a proportion of invariable sites and a subsampling frequency of 1,000. Posterior probability values >95 are indicated on the tree nodes. The black arrows indicate the sequences identified in this study. Scale bar indicates nucleotide substitutions per site.

CanineCV DNA was detected in a total of 8 animals, including 4/22 (18%) Eurasian badgers and 4/8 (50%) wolves, while no red foxes (0/232, 0%) tested positive. All positive wolf and Eurasian badger samples were obtained from intestines and spleens, respectively. Successful amplification and sequencing of partial Rep gene was obtained from 6/8 qPCR positive samples. Blast analysis revealed that 4 sequences (2 from Eurasian badgers and 2 from wolves) displayed a 92.8–98.5% nucleotide (nt) identity to CanineCVs detected in domestic dogs. By phylogeny, the wolf and badger sequences were found to cluster with CanineCV sequences obtained from domestic dogs and one Eurasian badger (Figure 2). Surprisingly, one sequence (145.20-5432) displayed 68.6% nt identity to a cyclovirus sequence from a domestic cat (GenBank accession no. KM017740), whilst the other (145.20-1329) was 79.4% identical to a cyclovirus sequence from bats (HQ738637); both cyclovirus sequences were identified from wolves. In the phylogenetic tree (Figure 3), the 4 CanineCV sequences from wild carnivores clustered with reference CanineCV sequences obtained from domestic dogs and wild carnivores, while both cyclovirus sequences clustered with a feline cyclovirus (KM017740) of possible bat origin.

All animals tested negative for CAdV-2 and CDV.

## Discussion

Over several decades, various efforts have been targeted toward the eradication of highly pathogenic viruses of domestic dogs, including the extensive use of vaccines against CPV, CDV, CAdV-1 (42). However, these viruses have persisted in dog populations as a consequence of immunization failures (43), introduction of infected animals from endemic areas (14, 32) or virus circulation in wildlife (6). Carnivores at the human-wildlife interface are known to influence the epidemiology of canine viruses (3, 44).

The present study shows that CAdV-1 and CanineCV are circulating in wild carnivore populations in Italy, whereas no sample tested positive for CAdV-2. There is scanty molecular data regarding CAdV-2 in wild carnivores. The preferred sample type for CAdV-2 diagnosis is represented by respiratory specimens, owed to the virus tropism (17, 45), although the virus has been recovered from digestive tract samples, including intestines, rectal swabs and tongue specimens (28, 46). In addition, CAdV-2 has likewise been detected in internal organs of infected animals, including the spleen of 5 wolves, thus accounting for a hematogenous dissemination of the virus (3, 47). In this study, the real circulation of CAdV-2 in the wild carnivore population could not be assessed since respiratory samples were not tested and only few wolves and Eurasian badgers were sampled. The virus has been reported in wolves with frequencies of infection ranging from 1.38 to 8.7% (3, 28, 48). A recent report from Italy detected CAdV-2 in tongue samples of two wolves, but at very low titers so that sequencing was not possible (28). There is only one report of CAdV-2 infection in a fox (1), although the low viral titers observed could not rule out a passive transit in the gut of contaminated material.

Of all animal species that were sampled, only foxes tested positive for CAdV-1. Foxes are known to be susceptible to CAdV-1 infection and harbor the virus for a long time, thus acting as potential reservoirs (1, 18). A molecular survey in European foxes reported an 18.8% detection rate of CAdV-1 (36), although a much larger proportion of the animals had antibodies, thus suggesting previous exposure (18, 48). The present study accounts for a much lower detection rate for CAdV-1 in Italian red fox populations. This finding could be related to sampling bias. It is possible that animals which were sick from the virus died in their dens undiscovered, while most of the animals which come into human landscapes are apparently healthy.

Conversely, circoviruses were detected in wolves and badgers but not in red foxes, which is in line with previous reports (6), suggesting a low susceptibility to circovirus infection of these animals with respect to other wild carnivores. Wolves are closely genetically related to domestic dogs, so it is not surprising that they exhibit the same virus susceptibility. On the other hand, badgers are known to host several viruses of dogs, including CPV, FPV and CDV (4, 18, 36). Detection of CanineCV in the spleens suggests that the Eurasian badgers were actively infected with the virus. Two wolves were simultaneously infected with CanineCV and cycloviruses since their intestines tested positive for CanineCV by qPCR (which does not recognize cyclovirus sequences) and for cycloviruses by PCR amplification and sequencing using a protocol able to amplify the Rep gene of all members of the family *Circoviridae*. The most likely explanation is that cycloviruses were present at high titers in the samples and were selected during PCR amplification and sequencing to the detriment of CanineCVs. Although there is previous evidence for CanineCV infecting wolves, the two cyclovirus sequences which were detected in the present study may suggest that these wild canids may harbor other types of circoviruses. Two CanineCV-positive wolves (145.20-1274 and 145.20-4615) from this study were co-infected with CPV-2b (4) (Table 1), which has been previously reported (6). As far as we know, this is the first report of cycloviruses in wolves. It is, however, difficult to conclude that the detected viruses were actively infecting the wolves, since residual ingesta contaminated by cyclovirus DNA derived from a prey could remain on the mucosal lining of the sampled intestine and extracted along with the tissue. There is need to further investigate wolves as potential hosts of cycloviruses by analyzing other tissues not potentially contaminated by ingesta or by performing techniques, such as in-situ hybridization and immunohistochemistry, able to detect the viral nucleic acid or antigens in the intestinal epithelium. The low relatedness of the cyclovirus sequences from this study with reference sequences indicates that these are likely prototypes of novel virus species.

A continuous surveillance in wild carnivores should be carried out in order to monitor the circulation in wildlife of viruses pathogenic for domestic carnivores and endangered wild species.

## Data Availability Statement

The datasets presented in this study can be found in online repositories. The names of the repository/repositories and accession number(s) can be found below: https://www.ncbi.nlm.nih.gov/genbank/, OL323110-OL323113; OL364172 OL364173 OL364174-OL638989; OL638987-OL638988.

## Ethics Statement

Ethical review and approval was not required for the animal study because this study was conducted on carcasses of animals found dead and submitted to routine necropsy procedures for diagnostic purposes.

## Author Contributions

LN: laboratory analyses, sequence analyses, and manuscript writing. GL: sequence analyses and manuscript writing. VV, CD, FP, and FB: laboratory analyses. CM, MC, SB, and GS: sample collection and processing. AC, GE, AP, VM, and CB: manuscript revision. ND: general supervision and manuscript revision. All authors contributed to the article and approved the submitted version.

## Funding

This study was supported by grants from the Italian Ministry of Health: Ricerca Corrente 2019 NGS e diagnostic a molecolare in Sanità Animale: Fast D2 (IZS AM 08/19 RC), recipient ND.

## Conflict of Interest

The authors declare that the research was conducted in the absence of any commercial or financial relationships that could be construed as a potential conflict of interest.

## Publisher's Note

All claims expressed in this article are solely those of the authors and do not necessarily represent those of their affiliated organizations, or those of the publisher, the editors and the reviewers. Any product that may be evaluated in this article, or claim that may be made by its manufacturer, is not guaranteed or endorsed by the publisher.
