# Supplementary material for: Detection and Genetic Characterization of Canine Adenoviruses, Circoviruses, and Novel Cycloviruses From Wild Carnivores in Italy
Source: Front Vet Sci. 2022 Mar 31;9:851987. doi: 10.3389/fvets.2022.851987 (PMC9010027; doi:10.3389/fvets.2022.851987)
Supplement: Supplementary file 1 [file Table_1.docx]

**Supplementary Table 1. Oligonucleotides used in detection and sequencing of canine adenoviruses, canine distemper virus, canine calicivirus and cycloviruses.**

| Oligonucleotide | Sequence | Specificity | Reference |  |
| --- | --- | --- | --- | --- |
|  |  |  |  |  |
| CAVF | 5′-AGTAATGGAAACCTAGGGG-3 | CAdV-1/2 | (37) |  |
|  |  |  |  |  |
| CAVR | 5′-TCTGTGTTTCTGTCTTGC-3 | CAdV-1/2 |  |  |
|  |  |  |  |  |
| CAV1-Pb | FAM-TCAATCGTCTCAACTAAATGCCGTG-BHQ1 | CAdV-1 |  |  |
|  |  |  |  |  |
| CAV2-Pb | TxR-TCAGTCATCTCAGCTCAATGCCGTG-BHQ1 | CAdV-2 |  |  |
|  |  |  |  |  |
| HEX-F | 5′-AAGTTTGCCGACCCTGTCTT-3 | CAdV1/2 |  |  |
|  |  |  |  |  |
| HEX-R | 5′-AGAAGAGCTGCGAGCCATAG-3 |  |  |  |
|  |  | CAdV1/2 |  |  |
| CDV-F | 5′-AGCTAGTTTCATCTTAACTATCAAATT-3 |  |  |  |
|  |  | CDV | [(38)](#_ENREF_19) |  |
| CDV-R | 5′-TTAACTCTCCAGAAAACTCATGC-3′ | CDV |  |  |
|  |  |  |  |  |
| CDV-Pb | FAM-ACCCAAGAGCCGGATACATAGTTTCAATGC-TAMRA | CDV |  |  |
|  |  |  |  |  |
| DogCV-forward | 5′-CTTGCGAGAGCTGCTCCTTATAT-3′ | CaCV | (26) |  |
|  |  |  |  |  |
| DogCV-reverse | 5′-CTCCACTTCCGTCTTCCAGTTC-3′ | CaCV |  |  |
|  |  |  |  |  |
| DogCV-probe | TCCGGAGATGACCACGCCCC | CaCV |  |  |
|  |  |  |  |  |
| CV-F1 | 5′-GGIAYICCICAYYTICARGG | CV | (31) | |
|  |  |  |  |  |
| CV-R1 | 5′-AWCCAICCRTARAARTCRTC | CV |  |  |
|  |  |  |  |  |
| CV-F2 | 5′-GGIAYICCICAYYTICARGGITT | CV |  |  |
|  |  |  |  |  |
| CV-R2 | 5′-TGYTGYTCRTAICCRTCCCACCA | CV |  |  |

CAdV-1, canine adenovirus type 1; CAdV-2, canine adenovirus type 2; CDV, canine distemper virus; CaCV, canine circovirus; CV, cyclovirus.
